# Complicated Grief: What to Expect After the Coronavirus Pandemic

**DOI:** 10.3389/fpsyt.2020.00489

**Published:** 2020-05-26

**Authors:** Camilla Gesi, Claudia Carmassi, Giancarlo Cerveri, Barbara Carpita, Ivan Mirko Cremone, Liliana Dell'Osso

**Affiliations:** ^1^ Department of Mental Health and Addiction, ASST Fatebenfratelli-Sacco, Milan, Italy; ^2^ Department of Clinical and Experimental Medicine, University of Pisa, Pisa, Italy; ^3^ Department of Mental Health and Addiction, ASST Lodi, Lodi, Italy

**Keywords:** complicated grief, bereavement, pandemic, COVID-19, natural disaster, ICU treatment, coronavirus outbreak, SARS-CoV-2

## Abstract

The COVID-19 pandemic is one of the worst public health crises in a century, with an expected amount of deaths of several million worldwide and an even bigger number of bereaved people left behind. Although the consequences of this crisis are still unknown, a significant number of bereaved people will arguably develop Complicated Grief (CG) in the aftermath of this emergency. If the current pandemic is unprecedented, the grief following the coronavirus outbreak is likely to share features with grief related to natural disasters and after Intensive Care Unit (ICU) treatment. The aim of this paper is to review the most prominent literature on CG after natural disasters, as well as after diseases requiring ICU treatment. This body of evidence may be useful for helping bereaved people during the acute phase of the COVID-19 pandemic and for drawing clinical attention to people at risk for CG.

## Introduction

At the time of writing this paper, over 690,000 people around the world have been infected with COVID-19, which has already caused more than 33,000 deaths. Although pandemic projections must be treated with caution at this early stage, it has been estimated that the global death toll from COVID-19 could reach as high as 15 million even in the best-case scenario (1).

Bereavement is a common human experience and grief represents the normal reaction to the death of a loved one. The vast majority of individuals who lose someone usually adjust over a period of six to 12 months and finally develop a new sense of normalcy in their life. But for others, this process becomes troublesome and prolonged. When people get stuck indefinitely in grieving, preventing them from processing the death and moving on with life, a condition known as complicated grief (CG) may eventually arise (2). CG is a chronic, impairing form of grief, distinctive from depression and post-traumatic stress disorder and other conditions that may follow the death of a loved one (3–6).

To the best of our knowledge, no studies examined CG in the aftermath of an epidemic or a pandemic. Still, the coronavirus outbreak shares several features with natural disasters that have been previously investigated with regard to CG. Moreover, people dying from COVID-19 usually do so after being admitted to ICU and remaining isolated from their loved ones until the end. This obviously poses an even heavier burden on the loss, but also makes it useful to keep in mind what may happen to bereaved people when the death occurs in such circumstances and what we must expect from their grieving in the aftermath of this global emergency.

Based on the above premises, the aim of this paper is to review the most prominent literature on CG after natural disasters, as well as after acute diseases requesting ICU treatment. This body of evidence may be useful for assisting bereaved people during the acute phase of the COVID-19 pandemic and for drawing clinical attention to people at risk for CG.

## Complicated Grief After Natural Disasters

The loss of a loved one during a natural disaster is especially traumatic and distressing, given that the death usually happens suddenly and unexpectedly (7). Similar to epidemics, natural disasters affect bereaved people in several ways other than causing a loved one's death. They often occur as massive traumatic events, putting others' and one's own life in danger and bringing about multiple kinds of losses at the same time, including losses in human lives, properties, income, and overall wealth. Most of the recent literature on natural disasters comes from earthquakes, tsunamis, and hurricanes (8–10). Prevalence estimates of CG among bereaved survivors evaluated up to 18 months after these types of disasters range from 9% to 80%, with the loss of a child or a spouse both being associated with CG symptoms (8, 11–13). Other factors that seem to be significantly associated with CG include extensive damage to homes (11, 14), being of the female sex, having fewer years of education (8, 13, 14), and lacking social support and social competence (8, 13) Interestingly, non-bereavement losses and PTSD symptoms have both been indicated as risk factors for CG during massive disasters (8, 15). The longest longitudinal study that evaluated the evolution of grief up to six years after an earthquake found that 41% of the bereaved had been following a resilient trajectory, characterized by low levels of CG, 48% had shown a recovering trajectory, with initially high symptoms of CG and a gradual decrease thereafter, and 11% had ended in a chronic trajectory, featuring high and unremitting levels of CG symptoms for as long as six years after the event. Interestingly, compared to other long-term studies evaluating CG after non-traumatic events/non-traumatic losses, the percentage of resilient subjects was not higher, which could be an indicator of the addictive burden of exposure to a traumatic event and a traumatic loss in increasing the risk of CG (16).

## Complicate Grief After Diseases Requiring ICU Treatment

Relatives of patients admitted to the ICU are at risk of psychological morbidity during and after the hospital stay (17–19). The ICU environment may be quite a traumatic experience for both patients and relatives: people are rolled in, often unable to communicate, hooked up to machines, ventilated or intubated. Visits are strictly controlled, and critical events, including death, may happen quickly, with no chance for relatives to be informed timely and to say goodbye. During the COVID-19 pandemic, most of the deaths have happened in the ICU after the development of an acute respiratory distress syndrome (20). In many cases, patients entered the ER, were taken into the ICU, and never came back. Meanwhile, in order to keep them isolated, no one could see them, nor could any loved one be there at the time of the death. Several news media outlets have been reporting that for those whose relatives got infected with COVID-19 and died in the ICU, the situation proved to be extremely traumatic.

Hospital and ICU mortality is especially associated with a high incidence of grief reactions, including post-traumatic stress disorder, anxiety, depression, and CG (19, 21–23). Prevalence estimates of CG in relatives of ICU decedents are quite variable, ranging from 5% to 52% as evaluated on average six months after the death (19, 22, 24, 25). In a study conducted among 50 family members of ICU inpatients, 46% of those whose relative died presented with both CG and PTSD after six months. CG was not associated with anxiety or depression at the time of the death, nor at one and six months after the event, or with sociodemographic variables, relationship to patient, or decision-making role preference (26). In a recent larger study involving 282 relatives of ICU decedents, the prevalence of CG at six months was as high as 52.1%, and CG symptoms were associated with the presence of depressive symptoms at three months. Interestingly, after 12 months, the proportion of assessable relatives with CG was unchanged, despite individual data revealing that approximately 20% of subjects developed CG between six and 12 months and a similar proportion recovered in the same period. Independent predictors of CG symptoms were either unchangeable variables, such as female sex, a relative living alone, and intensivist board certification before 2009, or potential targets for improvements, such as a patient's refusal of treatment, the death occurring while intubated, relatives present at the time of death, relatives unable to say goodbye to the patient, or poor communication between physicians and relatives (27). Another study comparing family members of patients who died in an ICU versus a non-ICU hospital setting found that uniquely distressing experiences in the ICU setting were primarily related to the patient's emotional status prior to death (anxiety, fear) and the families' perception that the patient received attention and support (19).

## Discussion

The COVID-19 pandemic represents one of the worst public health crises in a century, with an expected death toll of several millions of people worldwide. The prevalence of CG in the general population ranges from 2 to about 7% (10 to 20% among bereaved people, 28–31), but estimates have been shown to increase steeply in certain selected groups, such as older adults grieving the loss of a spouse, people grieving for unexpected/violent deaths, bereaved people exposed to natural or human-made disasters, or other traumatic events (7, 15, 32). Given the lack of studies investigating grief after epidemics of infectious diseases such as COVID-19, which represents an unprecedented phenomenon, we chose to review extant literature on CG after natural disasters and after death in the ICU because we found a few similarities between both scenarios and the ongoing COVID-19 outbreak.

Together with the loss of loved ones, natural disasters imply multiple losses, the closure of schools and facilities, the stopping of productive activities, and the reduction in services and supplies, which is mirrored in the COVID-19 epidemic. Moreover, people are placed in a traumatic environment, the exposure to which is multiplied by means of news media and social networks (33). On the other hand, deaths from COVID-19 often happen in the hospital and typically in the ICU, which is known to represent a highly traumatic experience for decedents' closest relatives.

Our review underlines that female sex is a risk factor for CG, not only in the general bereaved population but also among bereaved people of ICU decedents or in the context of natural disasters. While grieving a child or a spouse has been indicated as a risk factor for CG after a natural disaster, no differences have been found with regard to the kinship to the deceased among people who lose a relative in the ICU. However, available data have hitherto shown that the vast majority of deaths due to COVID-19 is among older males (20), which may suggest the need to draw a special clinical attention to widows and, to a lesser extent, to other relatives of COVID-19 deceased in the aftermath of the emergency. Both lines of literature provide evidence that the more the death is associated with PTSD-related symptoms, the higher the risk of concurrent CG might be. If the traumatic exposure during the pandemic cannot be easily modified, the traumatic burden of the ICU should be kept as low as possible. One of the greater issues of death in the ICU is that family members may be unable to say goodbye to their loved one, which was shown to be an independent risk factor for CG (19, 27). This is crucially important among the COVID-19 bereaved, who cannot access the ICU during the whole stay and for whom the separation from the decedent may contribute to feelings of yearning, anger, and bitterness over the death, ultimately leading to CG (27). In addition, it is likely that the COVID-19 bereaved might blame themselves for not having tried harder to see their loved one while in hospital and for not taking care of their relative's comfort and dignity during the hospital stay, which has been shown to impact on families' experience of the dying process (19, 27). For the same reasons, they could feel guilty about not being present at the time of death, although witnessing the death has been shown to raise the risk of CG in one previous study on bereavement after ICU stay (27). A further important issue encountered by families of people dying in the ICU and increasing the risk of CG is the poor communication with physicians (34), that may be arguably worse in the course of the COVID-19 pandemic due to a matter of protection against contagion as well as due to hospitals' overloading and staffing shortages of some countries. Again, both lines of literature highlight that greater levels of social support are correlated with better grief outcomes (13, 27). Unfortunately, differently from mass disasters occurring within a short period, the COVID-19 pandemic is a long-lasting condition, leading to durable changes in everyday life, and especially in social habits. This might make COVID-19 mourners particularly vulnerable, given that social distance has been claimed to be the most important action to prevent further spreading of the disease, a measure, however, that could increase the risk of unsuccessful grieving.

Lastly, it has to be noted that the ongoing COVID-19 pandemic bears the unprecedented circumstance that public funerals have become illegal in many countries due to national restrictions against gatherings and to cemeteries' overloading. Funeral and burial rituals are important for the affective adjustment of people grieving the loss of a loved one and mourners who drew comfort from planning and participating in the funeral were shown to achieve better outcomes in later grief (35). From this perspective, being prevented from holding a proper funeral for their loved ones might prevent COVID-19 mourners from gaining awareness of the reality of the death and from understanding and framing their loss, besides eliminating a significant important occasion of social support (35).

## Conclusive Remarks and Lines of Intervention

The COVID-19 crisis has united mental health stressors that have been separately evaluated in other contexts, but which have never been seen combined in one, unprecedented disaster happening on a global scale. This makes it very difficult to infer what is going to happen to people during and in the aftermath of this global event, unless we use previous data as a provisional proxy of such a new and unknown phenomenon.

COVID-19 is expected to cause an enormous death toll worldwide. Given that each death leaves up to five people grieving behind (36), and that the prevalence of CG is estimated at approximately 10% to 20% of bereaved people (28), the number of cases of CG following COVID-19 deaths may virtually reach the number of overall COVID-19 deaths in the upcoming months. The chance of lowering the psychological impact of bereaved people during the current pandemic is small, since only a few risk factors for CG can be manipulated by intervention. One concrete option is to safeguard the connectedness between relatives and COVID-19 inpatients and to improve the communication between medical staff and relatives, ensuring the latter is updated about the clinical evolution, and eventually allowing them into the ICU as the condition gets worse. A second focus of intervention might be to apply a shared approach to end-of-life, involving family members as surrogate decision makers. Indeed, besides representing an important component of high-quality end-of-life care in the ICU (37), the involvement of the relatives in treatment-limitation decisions was shown to correlate with decreased CG symptoms among survivors (27). Third, as living alone after the death has been found to be associated with the presence of CG symptoms, it could be crucial to provide bereaved relatives with effective social support. All this may obviously require an extra effort by the health system, that is already stressed and stretched beyond its readiness of facing the burden of the outbreak, at least in some regions (38). Still, mental health and social work departments and community organizations may share the burden of emergency and intensive care units and play a crucial role in providing emotional and practical support to the most vulnerable bereaved populations who display psychological symptoms. At the same time, mental health services could possibly be involved in surveying and alleviating critical stress levels among health workers of intensive care and emergency units, who might also be prone to developing a broad range of stress-related conditions, including CG following the death of colleagues and other staff members to coronavirus disease. Eventually, the implementation of specific services is warranted in the close future in order to convey tailored treatments to people developing CG after losing someone because of the pandemic. Given the frequent comorbidity between CG and both PTSD and depression, as well as the high traumatic burden of COVID-19, clinical attention should also be devoted, and treatment options tailored, to address all three conditions in the aftermath of the COVID-19 global emergency.

## Author Contributions

CG, GC, and LD'O conceived the work. IC and BC made the literature search and revision. CG, CC, and LD'O drafted the paper. CC and GC revised the work. All authors provided approval of the version to be published. 

## Conflict of Interest

The authors declare that the research was conducted in the absence of any commercial or financial relationships that could be construed as a potential conflict of interest.
